# Education alone is rarely enough: realising value from integrating simulation with quality improvement

**DOI:** 10.1186/s41077-026-00469-0

**Published:** 2026-07-15

**Authors:** Farrukh N. Jafri, Michael B. Light, Rafael E. Torres, Roger A. Edwards

**Affiliations:** 1 Department of Emergency Medicine, White Plains Hospital, White Plains, NY USA; 2 Department of Emergency Medicine, Rutgers New Jersey Medical School, Newark, NJ USA; 3 MGH Institute of Health Professions, Boston, MA USA; 4 Department of Emergency Medicine, Albert Einstein College of Medicine, Bronx, NY USA

**Keywords:** Patient simulation, Quality improvement, Patient safety, Translational simulation, In situ simulation, Donabedian model, PDSA cycles

## Abstract

The simulation community has invested heavily in educational rigour (validated debriefing methods, high-fidelity technology, faculty development), yet evidence linking simulation to sustained clinical improvement remains limited. We propose this gap exists not because simulation is ineffective, but because it is poorly positioned within organisational systems. Drawing on a decade of programmatic experience at a non-academic hospital, including multiple initiatives that did not produce sustained outcomes and subsequent methodological pivots, we argue that simulation achieves sustainable impact only when embedded within quality improvement infrastructure. Using the Donabedian model as a conceptual framework, we demonstrate that process-focused interventions (education, training) without corresponding structural supports (equipment, systems, protocols) produce transient change at best. Integrated approaches, where simulation serves as a quality improvement tool to identify system threats and monitor interventions through Plan-Do-Study-Act cycles and the SAFER-Matrix, produce measurable, sustained improvements. Evidence from in situ programmes across emergency departments and cardiac catheterisation laboratories, multi-site collaboratives, and AI-powered telehealth simulation demonstrates that this integrated approach succeeds where education alone falls short. We have formalised these principles in the Simulation-Quality Integration (SIM-QI) Framework, currently submitted for independent peer review, which provides a practical blueprint for embedding simulation within QI infrastructure. We present actionable implications for simulation educators, institutions, and researchers seeking to bridge the gap between educational excellence and clinical translation.

## Background

Simulation has become ubiquitous in healthcare education, yet a persistent challenge confronts the field: as Soong and Shojania observed, “education is an ineffective tool for quality improvement” [1]. This may seem provocative to simulation educators who have witnessed transformative learning experiences and measured significant improvements in knowledge, skills, and team performance. However, when viewed through the lens of translational outcomes, most simulation-based mastery learning studies demonstrate only T1 outcomes (improvements measured in educational laboratories) while evidence for T2 outcomes (improved clinical practice) and T3 outcomes (better patient results) remains uncommon [2]. A systematic review using the Kirkpatrick Model confirms that few healthcare simulation studies demonstrate or even evaluate effects at the highest levels of behaviour change and patient outcomes [3].

The field has responded to this gap by improving educational quality: more sophisticated debriefing methods, higher-fidelity mannequins, rigorous assessment rubrics, and extensive faculty development. These efforts are valuable and address necessary gaps, but they address the wrong problem. The assumption underlying these improvements is that better educational delivery will produce better clinical outcomes. Our experience suggests otherwise. The issue is less about inadequate implementation and more about poor positioning within organisational systems. When simulation exists outside quality improvement infrastructure, even exceptional education cannot produce sustained clinical translation.

This commentary addresses the specific challenge of why simulation that succeeds educationally often fails to produce sustained clinical impact. For programmes with purely educational goals (knowledge acquisition, skill development, learner competency), traditional simulation approaches remain appropriate and effective. For those who aim to improve clinical practice and patient outcomes, however, a fundamentally different mindset is required: one in which simulation is designed, delivered, and debriefed not primarily for individual learning but for systems improvement. We argue that simulation achieves sustainable impact only when embedded within quality improvement infrastructure, and we offer evidence from a decade of programmatic experimentation.

## Main text

### Origins: a simulation programme built on quality improvement

Our perspective is shaped by institutional context. White Plains Hospital is a non-academic hospital without residency programmes or medical students. While we conduct educational workshops for new hires and early-career nurses, the simulation programme was not conceived as an educational centre. From its inception, the programme was designed with an outcomes-based focus, asking how simulation could be leveraged in the translational space to improve clinical systems. The first author (FNJ) had already been conducting simulation independently during night shift downtime, using laboratory transport containers as improvised patients, with the explicit goal of improving clinical care rather than fulfilling educational requirements. When the hospital formalised its simulation centre, he was selected to direct it precisely because this work was already happening. The founding vision came from the director of the Emergency Department (author RET), who is now the Chief Quality Officer of the hospital, and who saw simulation as a natural extension of QI rather than a teaching tool that might someday prove useful for quality improvement. This orientation meant that our programme was embedded within quality infrastructure from the start, initially targeting high-risk, low-frequency procedures where system failures carry the greatest consequences. Simulation is the methodology, not the equipment; the principle that drives this work has never required high-fidelity mannequins or dedicated simulation centres. Yet having the right orientation did not immediately produce the right methodology.

### Lessons from a decade of intelligent failures

Despite this quality-focused foundation, our early programmes relied on the tools and frameworks the simulation field provided, and those tools were overwhelmingly educational. Over a decade of simulation programming, we designed and delivered multiple interprofessional training programmes using evidence-based educational methods: microdebriefing for paediatric resuscitation, TeamSTEPPS curricula for rapid response teams leveraging advocacy-inquiry, and virtual reality for procedural skills [4, 5]. Participants reported high satisfaction. Simulation scores improved. Yet we were unable to demonstrate sustained impact on clinical practice or quality outcomes. Our paediatric resuscitation programme trained teams from four departments using crisis resource management principles, with teamwork scores improving significantly (42.8% to 57.5%), yet concerns about team coordination during actual paediatric emergencies persisted. Our rapid response team training achieved improvements using validated rubrics with excellent inter-rater reliability, but continued communication breakdowns in real practice led to the intensive care unit assuming primary responsibility for rapid responses. Our Stop the Bleed research demonstrated that even high-technology interventions (simulation with feedback devices, virtual reality refreshers) produced short-term skill improvements that decayed within months. These programmes represent what Edmondson terms “intelligent failures”: experiments that did not produce hoped-for outcomes but generated essential learning [6]. Table 1 summarises this institutional evolution.

**Table 1:** Evolution of simulation-based quality improvement programmes at a single institution (2017–2025)

Year	Programme	Approach	Outcome	Donabedian
Phase 1: Education-Focused Simulation (2017–2019) — T1 outcomes only, no clinical translation				
2017	Sepsis Simulation	Online module + interprofessional team simulation	No change in order set utilisation; no impact on mortality	Process only
2018	Paediatric Resuscitation	Microdebriefing, CRM training across 4 departments	Simulation scores improved (42.8%→57.5%); continued clinical concerns	Process only
2019	Rapid Response Training	TeamSTEPPS + online modules + validated rubric (ICC 0.86–0.89)	Teams improved pre→post; ongoing communication concerns	Process only
2019	MCI Foundations	Logic model design; multi-site tabletop + simulation	Knowledge retained at 3 months; programme dissolved, no clinical impact	Process only
2019–21	Stop the Bleed	High-tech simulation, VR, feedback devices	Short-term gains; skill retention poor at 2–8 months	Process only
Phase 2: Methodological Pivot — In Situ Simulation as QI Tool (2019–2020)				
2019	Paeds Tracheostomy^*^	Multi-centre in situ; LST identification; equipment checklists	41 LSTs identified; equipment availability improved post-intervention	Structure + Process
2020	Paediatric Shock	In situ with QI tools; cause-and-effect diagrams	25 LSTs identified; interventions designed but follow-up prevented	Incomplete
Phase 3: Full QI Integration — PDSA + SAFER + Donabedian (2020–2024)				
2020–21	COVID In Situ Airway^*^	3 PDSA cycles; SAFER-Matrix; Donabedian framework	SAFER 10.94→4.71 (*p* = 0.017); structure-based threats improved (*p* = 0.001)	Structure + Process
2020–21	MISS-Q Multi-Site^*^	Same protocol across 5 EDs; 58 sims, 328 participants	LSTs/sim: 7.00→4.69 (*p* < 0.001); methodology replicated across sites	Structure + Process
2021	Cath Lab In Situ^*^	3 PDSA cycles; interdepartmental (ICU, Anaesthesia, Cards)	SAFER 5.37→1.00 (*p* = 0.011); communication threats linked to intubation time	Structure + Process
2021	Stop the Bleed + Cognitive Aids^*^	Training + point-of-care cognitive aids + priming	Combined approach: OR 12.67 (*p* < 0.001); structure enabled retention	Structure + Process
Phase 4: Scalable Translation — AI-Powered Simulation (2025)				
2025	AI Telehealth Simulation^*^	Validated rubrics + AI simulation + SPC monitoring; 468 real calls	19–37% improvement in real interactions; passive education produced no change	Structure + Process

^*^Denotes programmes using integrated QI methodology (PDSA cycles, SAFER-Matrix, Donabedian framework)

### A conceptual reframing: simulation as quality improvement tool

The Donabedian model provides a useful lens for understanding why education-focused simulation struggles to produce lasting change [7]. This framework conceptualises quality as the interrelationship among structure (the environment and resources where care occurs), process (the actions and behaviours during care delivery), and outcomes. Most simulation programmes focus predominantly on process, training people to behave differently, without explicitly addressing the structural context that enables or constrains those behaviours. Critically, this reframing is not simply about conducting simulations ‘in situ’ rather than in a laboratory. Moving location without changing the fundamental purpose of the simulation, from education to systems diagnosis, misses the point. The paradigm shift requires using the same language, playbook, and methods as any quality improvement project, but now with a powerful new tool. Simulation objectives should be framed as SMART aims tied to clinical outcomes, not as learning objectives tied to educational competencies. The question is not ‘What should learners know after this simulation?’ but ‘What is our system doing, and what does it need?’ Debriefing shifts correspondingly, from individual performance reflection to structured identification of latent safety threats, workflow failures, and structural gaps [8]. We should be candid about simulation’s limitations. It is an expensive resource that removes clinical staff from patient care responsibilities. The decision to deploy simulation should be weighed against simpler QI tools (chart review, direct observation, process mapping) that may diagnose a system problem with less disruption. Simulation’s unique value lies in its ability to probe how systems respond under controlled conditions that would be unsafe or impractical to create with real patients, and to surface failure modes that static observation cannot reveal. Where that diagnostic function is not needed, simpler tools should be preferred. Viewed through the lens of the Quintuple Aim (better outcomes, better experience, lower costs, clinician well-being, and health equity) [9], simulation must justify its use not only by improving outcomes but by doing so in a manner that is sustainable and does not impose undue burden on the workforce it is meant to support.

Our Stop the Bleed research provides a particularly clear illustration. Training school personnel in haemorrhage control with simulation and feedback devices produced significant short-term improvement, but skill retention at two to eight months was poor in both intervention and control groups [10]. Virtual reality refresher training showed no improvement over control [11]. Education alone, even technologically enhanced education, was insufficient. However, when we added a structural element, a mobile phone application providing cognitive support at the point of care, correct tourniquet placement improved dramatically (62.5% vs. 30.0%, *p* = 0.01). A subsequent randomised trial of 346 participants found that cognitive aids combined with prior training produced the strongest effects (OR 12.67, *p* < 0.001) [12]. The same skill, similar populations, but integration of structure and process produced dramatically different results.

This insight aligned with a principle from Lean methodology: going to the ‘gemba’, the Japanese term for “the actual place.” Quality improvement requires observing work where it occurs and addressing system-level factors that enable or inhibit performance. Traditional simulation, conducted in laboratories removed from clinical spaces, misses this opportunity. In situ simulation, conducted in the actual care environment using real equipment and existing teams, is inherently a ‘gemba’ activity, exposing latent safety threats that would never surface in a simulation centre: equipment that cannot be located, workflows that break down under pressure, communication systems that fail when needed most.

This reframing aligns with what Brazil and colleagues have termed “translational simulation”, simulation designed to explore and improve systems rather than solely develop individual competency [13, 14]. Despite compelling articulation of this paradigm, translational simulation remains “barely perceptible in most healthcare quality and safety practice” [14]. We operationalised this vision by repositioning simulation from intervention to diagnostic and monitoring tool, integrating it with quality improvement infrastructure: the Institute for Healthcare Improvement’s Model for Improvement with Plan-Do-Study-Act cycles for iterative testing, the SAFER-Matrix for prioritising threats by likelihood and scope of harm, run charts for distinguishing true improvement from random variation, and driver diagrams for connecting interventions to outcomes [15]. We have since formalised this approach in the Simulation-Quality Integration (SIM-QI) Framework, which maps simulation onto three phases of QI work (discovery, intervention, and sustainment) and explicitly positions diagnostic simulation before any intervention design, resisting the default organisational instinct to begin with training (Jafri FN, Johnson K, Yang CJ, Edwards RA: The Simulation-Quality Integration (SIM-QI) Framework: a practical blueprint for embedding healthcare simulation into quality improvement infrastructure, submitted).

### Evidence for the integrated approach

Our methodological pivot came during COVID-19, when our emergency department identified critical gaps in airway management for patients with SARS-CoV-2. Rather than designing a training programme, we implemented a structured quality improvement initiative using in situ simulation as the primary diagnostic tool [16]. Three PDSA cycles of ten simulations each were conducted over nine months. Latent safety threats were categorised, scored using the SAFER-Matrix, and addressed through cause-and-effect and driver diagrams. The median SAFER score decreased from 10.94 in cycle one to 4.71 in cycle three (*p* = 0.017). Critically, the Donabedian framework revealed why some threats improved while others persisted: structure-based threats (equipment location, PPE availability, cart organisation) showed significant improvement (1.28 decrease per cycle, *p* = 0.001), while process-based threats (communication workflows, role designation) did not improve until we added corresponding structural supports, specifically an AI-enabled huddle system providing protected time for team communication. Structure enabled process; process alone was insufficient. This was not a matter of conducting more simulations in the same clinical space; it was a fundamental redesign of what the simulations were intended to accomplish: discovering system vulnerabilities rather than achieving clinical responses.

We replicated this in the cardiac catheterisation laboratory, where acute airway emergencies are rare but catastrophic [17]. Eleven simulations across three PDSA cycles produced SAFER score decreases from 5.37 to 1.00 (*p* = 0.011). Communication-related threats directly correlated with time to definitive airway, with each additional threat adding 35 s to intubation time (*p* = 0.037), linking process measures to clinical outcomes in a way education-focused simulation rarely achieves. The bidirectional relationship between structure and process was striking: the department purchased an automated mechanical compression device (a structural intervention), but without corresponding processes (battery protocols, role designation, deployment algorithms), the equipment did not achieve its intended benefit. Structure without process was as ineffective as process without structure.

To test replicability, we formed a multi-site collaborative across five emergency departments, both academic and community, urban and suburban [18]. Using the same protocol, 58 simulations with 328 unique participants demonstrated latent safety threats per simulation decreasing from 7.00 to 4.69 (*p* < 0.001). Equipment-related threats showed the most consistent improvement (3.00 to 1.46, *p* < 0.001). The methodology transferred across diverse institutional contexts, suggesting the approach is generalisable rather than institution-dependent. Importantly, each of these programmes involved distinct clinical teams across different departments and institutions, not repeated simulations with the same cohort. The consistency of results across paediatrics, the emergency department, cardiac catheterisation, and five separate hospitals reinforces that the methodology, not familiarity or relationship-building with a particular team, drives the observed improvements.

Most recently, we tested whether these principles could scale with artificial intelligence. Our telehealth transitional care programme had established validated communication rubrics and statistical process control monitoring (structure), but quality improvement monitoring of 468 real patient calls revealed no meaningful change from passive education: rubric distribution, online modules, and didactic sessions (process) [19] (Jafri FN, Elsener M, Pulido K, Kumar A, Kardong-Edgren S, Edwards RA: Implementation of artificial intelligence–powered patient simulation for communication training in a telehealth transitional care program: a quality improvement study, submitted). Performance remained within expected variation, indicating random fluctuation rather than true improvement. Only after replacing passive education with active deliberate practice through AI-powered patient simulation did sustained improvement occur: total rubric scores improved 19–37% across all three clinical pathways (all *p* < 0.001), confirmed as true process improvement by statistical process control analysis. The quality of the process intervention mattered as much as its presence.

### Implications for the field

Notable successes in the broader literature reinforce these principles. Simulation-based team training reduced stroke door-to-needle times from 27 to 13 min while simultaneously reducing 90-day mortality, achieved through combined protocol revision and simulation training; the authors concluded that “the combination of protocol changes and simulation-based training eased the process of clinical implementation” [20]. Structure and process working together.

For simulation educators, this evidence suggests positioning simulation as a quality improvement tool rather than a standalone educational intervention. The Donabedian model should be applied prospectively: before designing training, identify the structural supports needed to enable desired behaviours. Communication training without communication systems will fail; equipment training without accessible equipment will decay. Embrace in situ simulation as going to the ‘gemba,’ and integrate quality improvement methods (PDSA cycles, the SAFER-Matrix, driver diagrams) to transform simulation from episodic training to continuous improvement. The SIM-QI Framework, currently submitted for independent peer review, operationalises this approach through a structured discovery phase that resists the instinct to begin with education, instead using diagnostic simulation to understand the system before designing any intervention (Jafri FN, Johnson K, Yang CJ, Edwards RA: The Simulation-Quality Integration (SIM-QI) Framework: a practical blueprint for embedding healthcare simulation into quality improvement infrastructure, submitted). Projects begin with SMART aims tied to clinical outcomes rather than simulation-specific learning objectives, using the same QI playbook any improvement team would use. When education is identified as a genuine need through the discovery process, active learning strategies are deployed alongside structural supports; when education is not the issue, the team proceeds directly to structural and process changes. This distinction, between simulation as a default educational response and simulation as a diagnostic tool that may or may not reveal an educational need, represents the core mindset shift.

### Earning a seat at the quality table

For institutions, integrating simulation into quality committee structures requires more than an invitation; it requires sustained demonstration of value through outcomes that leadership already monitors. Our path was neither linear nor easy. Simulation is resource-intensive and time-consuming, and it had not consistently demonstrated clinical value at our institution or in the broader literature, making leadership buy-in an ongoing negotiation rather than a settled question. The COVID-19 airway programme proved pivotal: when we demonstrated statistically significant reductions in latent safety threats using methods the quality committee understood (PDSA cycles, run charts, SAFER scores), the perception of simulation shifted from ‘educational exercise’ to ‘systems improvement tool.’ This was not achieved by running programmes and reporting results; it was achieved through systems redesign, going to the ‘gemba,’ and presenting findings in the language of quality improvement rather than the language of education.

Today, the individuals directing simulation sit on several quality committees, and our simulations have extended into environmental services for patient experience and room turnaround, de-escalation practices in our new psychiatric emergency unit with interdisciplinary staff, expanded paediatric resuscitation work, and updating our code stork protocols for deliveries in the ED. This positioning is not ceremonial; it means the simulation team sees the institutional priorities in real time, understands what metrics leadership is trying to move, and can ask the right question: not only ‘Can simulation help?’ but ‘Should simulation be used here, or would a less resource-intensive approach achieve the same result?’ Simulation may be triggered by a sentinel event, but it can also be driven by organisational priorities: a hospital struggling with sepsis bundle compliance, rising readmission rates, or patient experience scores may benefit from simulation-based systems diagnosis. This dual lens, reactive (trigger events) and proactive (organisational priorities), positions simulation as a strategic resource rather than a reflexive response. However, demonstrating value should not become an end in itself at the expense of responsiveness. The pursuit of measurable outcomes should not prevent simulation services from adapting to the complex and dynamic needs of the organisation. The demonstration of value and the outcomes measured are themselves a negotiation requiring collaboration and frequent re-evaluation with institutional stakeholders. Simulation services must remain responsive to shifting organisational priorities, and the metrics by which value is judged should evolve as those priorities change.

### Building capacity in resource-constrained settings

Our experience suggests the resource barrier is as much about leadership mindset and perception of value as it is about cost. Our institution did not require large startup costs. The earliest programmes used laboratory transport containers as improvised patients during night shift downtime. We used low-fidelity mannequins throughout the programmes described in this commentary. We scaled simulation with artificial intelligence when human standardised patients were impractical [19]. The critical investment was not equipment but organisational commitment. Simulation staff have some protected time, but it was insufficient for the scope of programmes we conducted; as primarily clinical staff, we designed and delivered simulations during our own clinical shifts. What mattered was willingness to act on findings and a shared expectation between simulation and quality leadership about what ‘success’ would look like. This negotiation of shared expectations was essential. Rather than promising transformative outcomes from the outset, we began with modest, achievable aims: identifying latent safety threats in a single clinical area, demonstrating that they could be reduced through iterative PDSA cycles, and presenting those results using methods quality leaders recognised. Each successful cycle built credibility for the next.

We recognise that our experience reflects simulation educators transitioning to quality improvement methodology. An alternative pathway, quality and safety staff learning simulation techniques, may prove equally viable but entails a substantial investment in simulation expertise. The time required to develop competency in scenario design, facilitation, and debriefing is considerable. We believe the responsibility falls primarily on simulation practitioners invested in translational simulation to demonstrate the value proposition convincingly enough that quality leaders see simulation as worth that investment.

For researchers, this body of work suggests moving beyond educational laboratory outcomes. The field has abundant evidence that simulation improves performance in controlled settings. What is needed is translational evidence linking simulation to clinical practice change and patient outcomes, and this requires embedding simulation within quality improvement frameworks that measure these endpoints. Equally important is reporting structural and process elements alongside educational methods. A paper describing debriefing techniques without describing the system context provides incomplete guidance for replication. Researchers should track sustainability over time, not just immediate post-intervention scores. Our Stop the Bleed research showed skills decay within months when only process interventions are applied.

## Conclusions

Simulation embedded within quality improvement infrastructure can be transformational, identifying threats invisible to other methods, enabling rapid-cycle testing of interventions, and producing sustained improvements in clinical practice. This requires more than relocating simulation to clinical spaces; it requires a paradigm shift in how simulation is designed, delivered, and debriefed, with systems improvement rather than individual learning as the primary objective. Simulation as a standalone educational technique, however rigorous, will continue to produce transient gains that fade without structural support. The field has robust educational methods. What is needed is the organisational commitment to position simulation as a quality improvement tool: to go to the ‘gemba,’ to ensure structure and process are developed together, and to complete the cycles necessary to demonstrate and sustain impact. This is achievable at our community hospital without residents, medical students, or dedicated research infrastructure, and it is perhaps even more achievable at academic institutions where robust simulation centres already exist. We validated this approach across diverse clinical contexts: emergency departments, cardiac catheterisation laboratories, inpatient units, telehealth programmes, and five independent institutions. The SIM-QI Framework provides a practical blueprint for this integration (Jafri FN, Johnson K, Yang CJ, Edwards RA: The Simulation-Quality Integration (SIM-QI) Framework: a practical blueprint for embedding healthcare simulation into quality improvement infrastructure, submitted). The question is not whether simulation works to achieve important outcomes, but whether we will position it to reach its potential.

## Data Availability

Data sharing is not applicable to this article as no datasets were generated or analysed during the current study. Data from the individual studies referenced are available from the corresponding author on reasonable request.

## Abbreviations

AI: Artificial intelligence
COVID-19: Coronavirus disease 2019
CRM: Crisis resource management
ED: Emergency department
ICC: Intraclass correlation coefficient
LST: Latent safety threat
MCI: Mass casualty incident
OR: Odds ratio
PDSA: Plan-Do-Study-Act
PSI: PEARLS for Systems Integration
PPE: Personal protective equipment
QI: Quality improvement
SAFER: Scoring Assessment For Enhancement of Reporting
SARS-CoV-2: Severe acute respiratory syndrome coronavirus 2
SIM-QI: Simulation-Quality Integration
SPC: Statistical process control
TeamSTEPPS: Team Strategies and Tools to Enhance Performance and Patient Safety
VR: Virtual reality
